# Influence of frosted haptics on rotational stability of toric intraocular lenses

**DOI:** 10.1038/s41598-021-94293-3

**Published:** 2021-07-23

**Authors:** Ruriko Takaku, Shinichiro Nakano, Masaharu Iida, Tetsuro Oshika

**Affiliations:** 1 Division of Ophthalmology, Ryugasaki Saiseikai Hospital, Ibaraki, Japan; 2 Department of Ophthalmology, Faculty of Medicine, University of Tsukuba, 1-1-1 Tennoudai, Tsukuba, Ibaraki 305-8575 Japan

**Keywords:** Eye diseases, Lens diseases

## Abstract

We investigated the unfolding property and rotational stability of a new toric intraocular lens (IOL); TECNIS toric II (toric-II, ZCW, Johnson & Johnson) that is an improved version of TECNIS toric IOL (toric-I, ZCV). Both IOLs are based on an identical platform, except for the frosted haptics with toric-II IOL. The study consisted of two parts; experimental study and clinical, retrospective, case series. Experimental study indicated that the overall time from IOL ejection to unfolding to 11 mm was significantly shorter with toricII than toric-I IOLs (p = 0.032), due to the earlier separation of the haptics from the optic with toric-II IOL. Clinical study included 131 eyes of 99 patients who had undergone phacoemulsification and toric IOL implantation. At 3 months postoperatively, toric-II IOL showed significantly better rotational stability than toric-I IOL, including smaller residual manifest astigmatism (p = 0.018), less amount of axis misalignment from the intended axis (p = 0.04), lower incidence of misalignment > 10º (p = 0.0044), and less degree of prediction errors (p = 0.043). Postoperative uncorrected distance visual acuity tended to be better in the toric-II than in the toric-I groups, with marginal statistical difference (p = 0.057). TECNIS toric II IOL with the frosted haptics showed significantly better rotational stability than its predecessor, probably due to quicker unfolding and greater friction with the capsular bag.

## Introduction

In patients undergoing cataract surgery, pre-existing corneal astigmatism can be surgically treated with adjustment of incision size and location, addition of peripheral corneal relaxing incision, performance of full thickness corneal incision on the steepest meridian, or implantation of a toric intraocular lens (IOL). Among these, implantation of a toric IOL is widely incorporated into the modern cataract surgery as the most predictable and feasible treatment option to reduce visually significant corneal astigmatism.

Misalignment of toric IOLs is caused by incorrect IOL placement at the time of surgery, occasionally by preoperative miscalculation, but it mostly occurs due to spontaneous rotation shortly after implantation^1^. Previous studies demonstrated that postoperative rotational stability varies across different model of toric IOLs^2–7^, indicating that the design, material, and surface finish of the IOL, especially haptics, should be among the important factors affecting postoperative rotational stability of toric IOLs. One study revealed that modification of the haptics of a toric IOL, by increasing overall length, reducing unfolding time, and introducing texture processing to the surface of haptics, significantly reduced postoperative axis misalignment^8^.

TECNIS toric IOL of Johnson & Johnson Vision Care, Inc. (Santa Ana, CA) has been regarded to have relatively poor rotational stability with greater amount of misalignment^3,4,6,7^ and higher rate of repositioning surgery^5,7^. The manufacturer recently modified this toric IOL to be a new version, TECNIS II, by introducing frosted haptics, which are aimed to offer more surface texture and friction between the lens haptics and the capsular bag. Such modification is likely to play a role in the rotational stability of toric IOLs, but no study has yet proven that assumption. The current experimental and clinical studies were aimed to compare TECNIS toric-I (conventional model) and toric-II (new model) IOLs, in terms of unfolding property and surgical outcomes.

## Methods

### Experimental study

**Figure 1:**
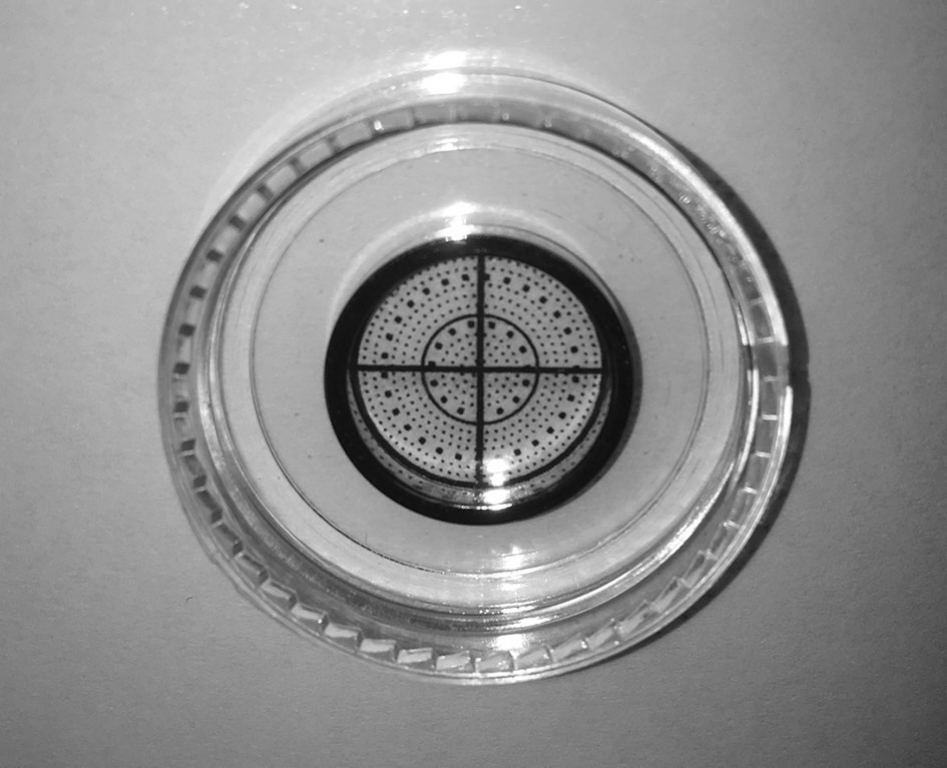
The petri dish filled with balanced salt solution and polar grid of concentric circles with 1-mm steps placed at the bottom. The unfolding process of toric intraocular lenses after ejection from the injector was video-recorded and analyzed.

The unfolding time was compared between the two models of toric IOLs with same power (+ 20.0 diopters) and toricity (model 150). Five IOLs of each model were used. The experiment was carried out at room temperature of 24.0 °C. The IOLs were set in the injector cartridge (Unfolder Platinum injector, Johnson & Johnson Vision Care, Inc.) with an ophthalmic viscoelastic device (sodium hyaluronate 1.0%, Healon, Johnson & Johnson Vision Care, Inc.). The IOLs were ejected in a petri dish filled with balanced salt solution and polar grid of concentric circles with 1-mm steps placed at the bottom (Fig. 1). The unfolding process of the IOLs were video recorded. The time (seconds) from the ejection of IOL to (1) initial movement of the haptics, (2) complete separation of the haptics from the optic, and (3) unfolding of IOL to 11.0 mm in length was measured. In each group, 25 measurements were done with five IOLs repeatedly used in rotation.

### Clinical study: patients

The clinical study included 131 eyes of 99 patients who had been treated with phacoemulsification and toric IOL implantation from July 2019 to July 2020. Toric IOLs were indicated for eyes having corneal regular astigmatism of 0.75 diopter (D) or more. The eyes with ocular or systemic disorders that can influence surgical outcomes were not included in the subjects. When there were intraoperative complications that would affect IOL stability, those eyes were excluded from the study. The patients were followed up for at least 3 months after surgery. An informed consent in a written form was obtained from each patient before surgery. The study adhered to the tenets of the Declaration of Helsinki, and the institutional review board of Ryugasaki Saiseikai Hospital approved the study protocol.

### Intraocular lenses

Two models of TECNIS toric IOLs were used; toric IOL (toric-I IOL, ZCV) and toric II IOL (toric-II IOL, ZCW), which are based on a similar platform including material, refractive index, design, configuration, and mechanical properties, except for the finish of side surface of the haptics. The toric-I IOL has smooth surface finish, while the toric-II IOL has frosted haptics. The common IOL specifications include overall diameter of 13.0 mm, optic diameter of 6.0 mm, biconvex optic with anterior toric aspheric surface and square optic edge, ultraviolet-blocking hydrophobic acrylic material, and refractive index of 1.47 at 35 °C.

### Surgery

All cased were operated on by a single surgeon (SN) using standard phacoemulsification and IOL implantation through a 2.2-mm corneoscleral incision located at the 12 o’clock. Anterior capsulorhexis of approximately 5.0 mm in diameter was created and the IOL was implanted into the capsular bag with an injector. For the axial alignment of a toric IOL, the digital image-guide system (VERION, Alcon Laboratories, Inc., Fort Worth) was used, which consists of a measurement module and digital marker^1^. Before surgery, the measurement module recorded high-resolution color reference images of patient’s eye, which were transferred to the digital marker. Based on multiple reference points on the conjunctiva and limbus, a digital overlay of the imported preoperative image and live-surgery image were created. The influence of eye movements and cyclotorsion is suppressed by the eye-tracking navigation system, and the targeted alignment axis of a toric IOL is precisely projected in the right ocular of the surgeon’s microscope.

### Examinations

Before surgery, axial length and corneal curvature were measured with IOLMaster 500 (Carl Zeiss, Germany) and IOL power was determined with the SRK/T formula. The toric IOL model and targeted placement axis were calculated using the designated manufacturer’s online calculator programs.

Corneal astigmatism, manifest astigmatism, and uncorrected distance visual acuity (UDVA) were measured before and 3 months after surgery. Using the swept-source anterior segment optical coherence tomography (AS-OCT, CASIA, Tomey Corp., Nagoya, Japan), toric axis misalignment was measured at 3 months postoperatively. The toric IOL analysis tool equipped with the AS-OCT demonstrates an image of the anterior segment with an overlapped green linear marker that can be rotated on a fulcrum automatically cantered on the corneal apex^9^. The linear marker is aligned parallel to the line connecting the marking dots of the toric IOL, and the direction of the alignment is expressed in angle degrees. The corneal topography obtained from the same scan is shown along with the power of steeper and flatter meridians, their axes, as well as the amount of the resulting topographic cylinder. The misalignment of the toric IOL axis from the intended orientation is expressed by the difference in degrees between the topographic axis and the value calculated for the linear marker^9^. The accuracy and repeatability of AS-OCT measurements have been reported elsewhere^10–12^.

The accuracy of astigmatism correction by toric IOLs was evaluated with double-angle plots of refractive astigmatism prediction errors being evaluated with centroids, standard deviation of the centroids, and 95% confidence ellipses of the prediction errors^13^. The percentage of eyes with refractive astigmatism prediction errors less than or equal to 0.25D, 0.50D, and 1.0D were also calculated.

### Statistical analysis

Numerical data are expressed as mean ± standard deviation. Statistical comparisons between two groups were performed using the Mann–Whitney U test. The categorical data were compared between groups with the χ^2^ test or the Fisher’s exact test. Statistical analysis was performed using SPSS Statistics for Windows software (version 26, IBM Corp., Armonk, NY, USA). A p-value of less than 0.05 was considered statistically significant.

## Results

### Experimental study

**Figure 2:**
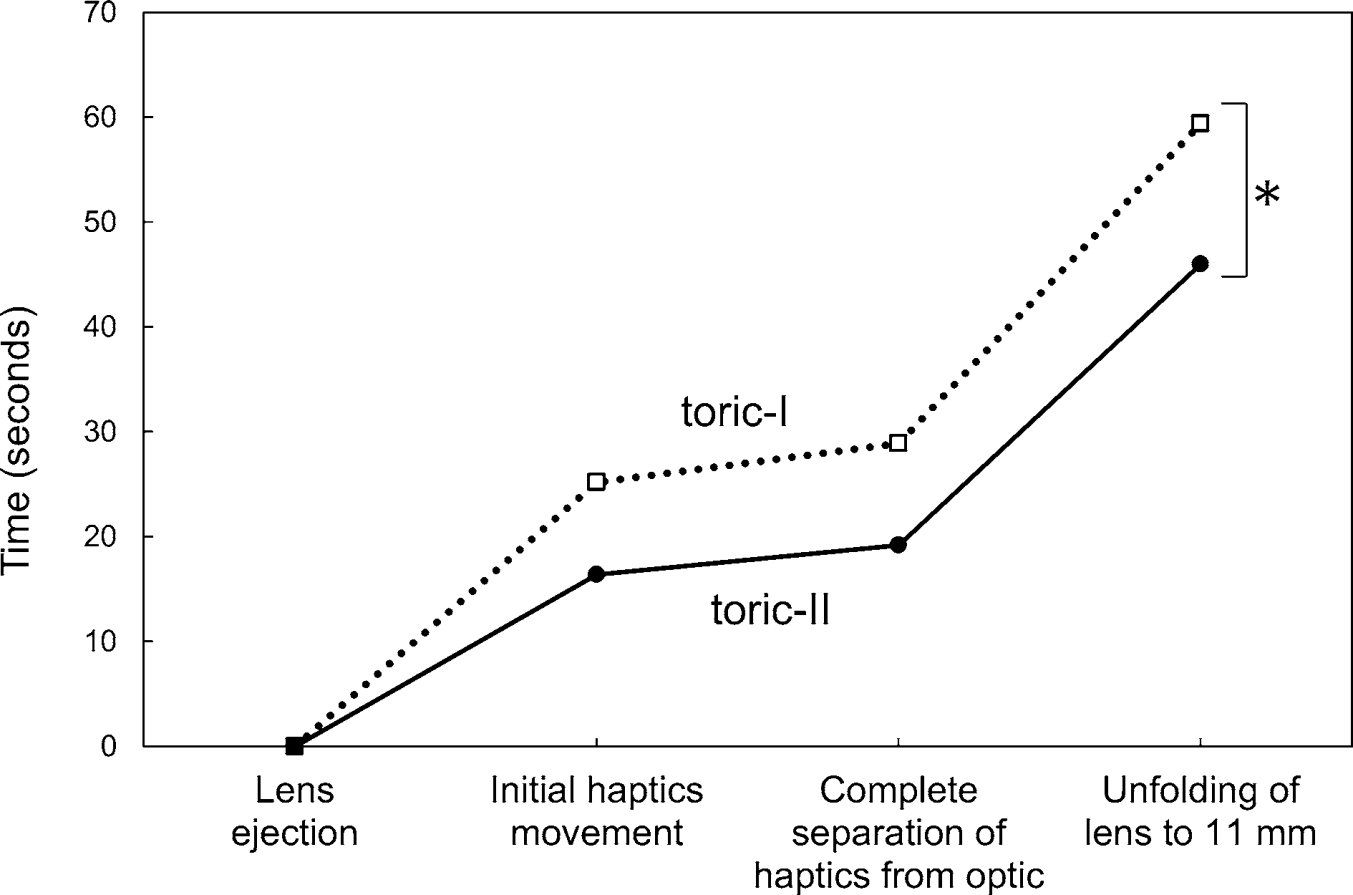
Elapsed time for each unfolding step of toric intraocular lenses. After the lens was ejected from the injector, the haptics started to move earlier with toric-II than with toric-I IOLs. Thereafter, the unfolding process of both toric IOLs were similar and the time course was parallel. The overall time from IOL ejection to unfolding to 11 mm was significantly longer with toric-I than with toric-II IOLs (p = 0.032).

The elapsed time for each step during lens unfolding is shown in Fig. 2 (supplementary video). After the IOL was ejected from the injector, the haptics of toric-II IOL started the initial movement earlier than those of toric-I IOL, though statistical difference remined marginal (p = 0.086). Thereafter, the unfolding process of both toric IOLs was similar and the time course was parallel. The time from initial haptics movement to complete separation of the haptics from the optic (p = 0.186) and from complete separation to unfolding to 11 mm in length (p = 0.554) was not statistically different between groups. The cumulative time from IOL ejection to unfolding of IOL to 11 mm was significantly longer with toric-I IOL than with toric-II IOL (p = 0.032).

### Clinical study

Seventy eyes received toric-I IOL from July 2019 to January 2020, and 61 eyes received toric-II IOL from January to July 2020. Their preoperative characteristics are shown in Table 1. No significant differences were found in the baseline characteristics of patients between the groups.

**Table 1:** Patient characteristics.

	Toric-I	Toric-II
Eyes (n)	70	61
Age	72.5 ± 9.4	71.4 ± 7.3
Male/female	33/37	23/38
Axial length (mm)	23.5 ± 0.9	23.3 ± 0.8
Preoperative corneal cylinder (D)	1.21 ± 0.4	1.32 ± 0.4
IOL power (D)	18.8 ± 4.7	18.8 ± 4.6
Target refraction (D)	− 0.57 ± 0.88	− 0.60 ± 0.95
Type of preoperative corneal cylinder: WTR/ATR/oblique	14/51/5	13/42/6
Model of toric IOLs: 150/225/300/375	31/26/13/0	20/25/12/4

Mean ± standard deviation; *IOL* intraocular lens, *D* diopters, *WTR* with-the-rule astigmatism (steep corneal cylinder axis was between 60° and 120°), *ATR* against-the-rule astigmatism (steep corneal cylinder axis was between 0° and 30° or 150° and 180°), *oblique* oblique astigmatism (steep corneal cylinder axis was between 30° and 60° or 120° and 150°).

Repositioning surgery to correct major axis misalignment was performed in 2 eyes (2/71, 2.8%) in the toric-I IOL group, while no eye (0/61, 0%) required reorientation surgery for axis misalignment in the toric-II IOL group. In the 2 eyes that underwent axis repositioning surgery, misalignment of 42° and 37° were surgically corrected 14 days after the primary cataract surgery, and the final orientation of toric axis was 0° and 2° from the targeted axis, respectively. In these eyes, the data immediately before the realignment procedure was used in the following analyses.

**Figure 3:**
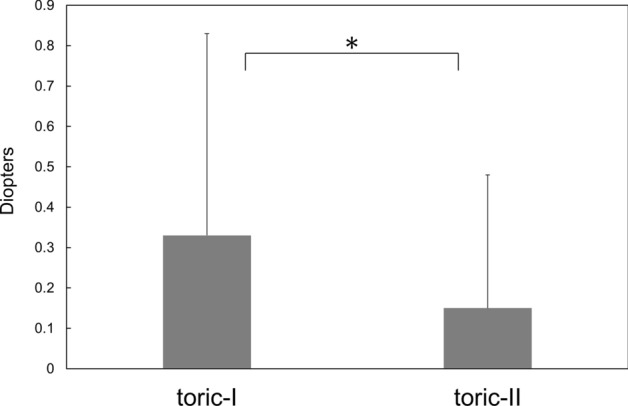
Amount of residual manifest astigmatism was significantly smaller with toric-II than with toric-I IOLs (p = 0.018).

**Figure 4:**
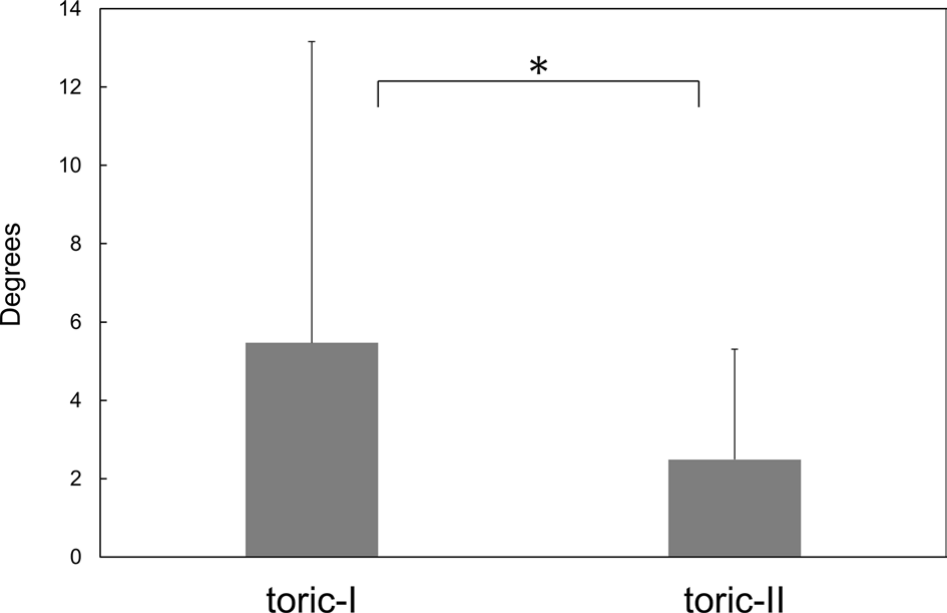
Amount of axis misalignment from the intended axis was significantly smaller with toric-II than with toric-I IOLs (p = 0.04).

**Figure 5:**
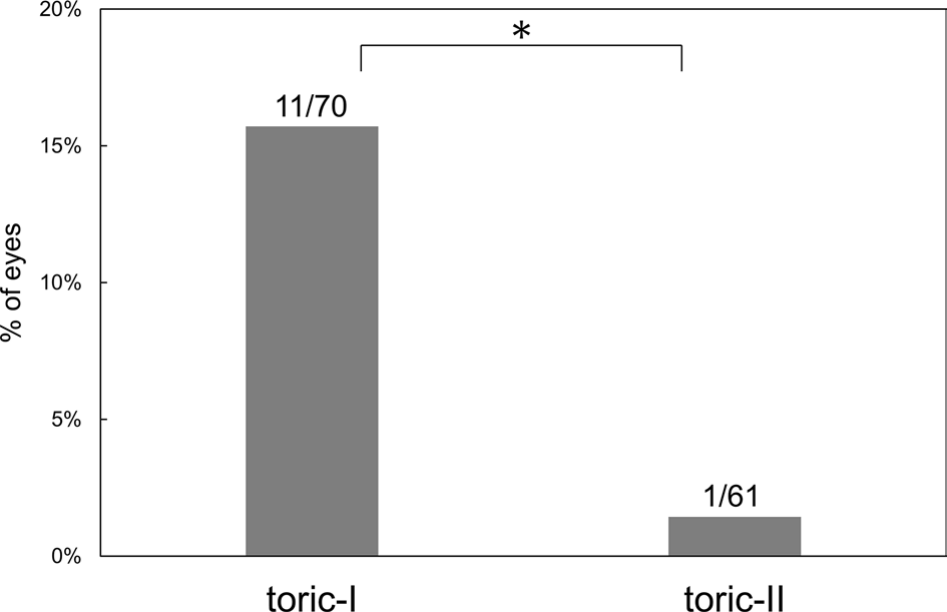
Percentage of eyes with misalignment > 10° was significantly smaller with toric-II than with toric-I IOLs (p = 0.0044).

**Figure 6:**
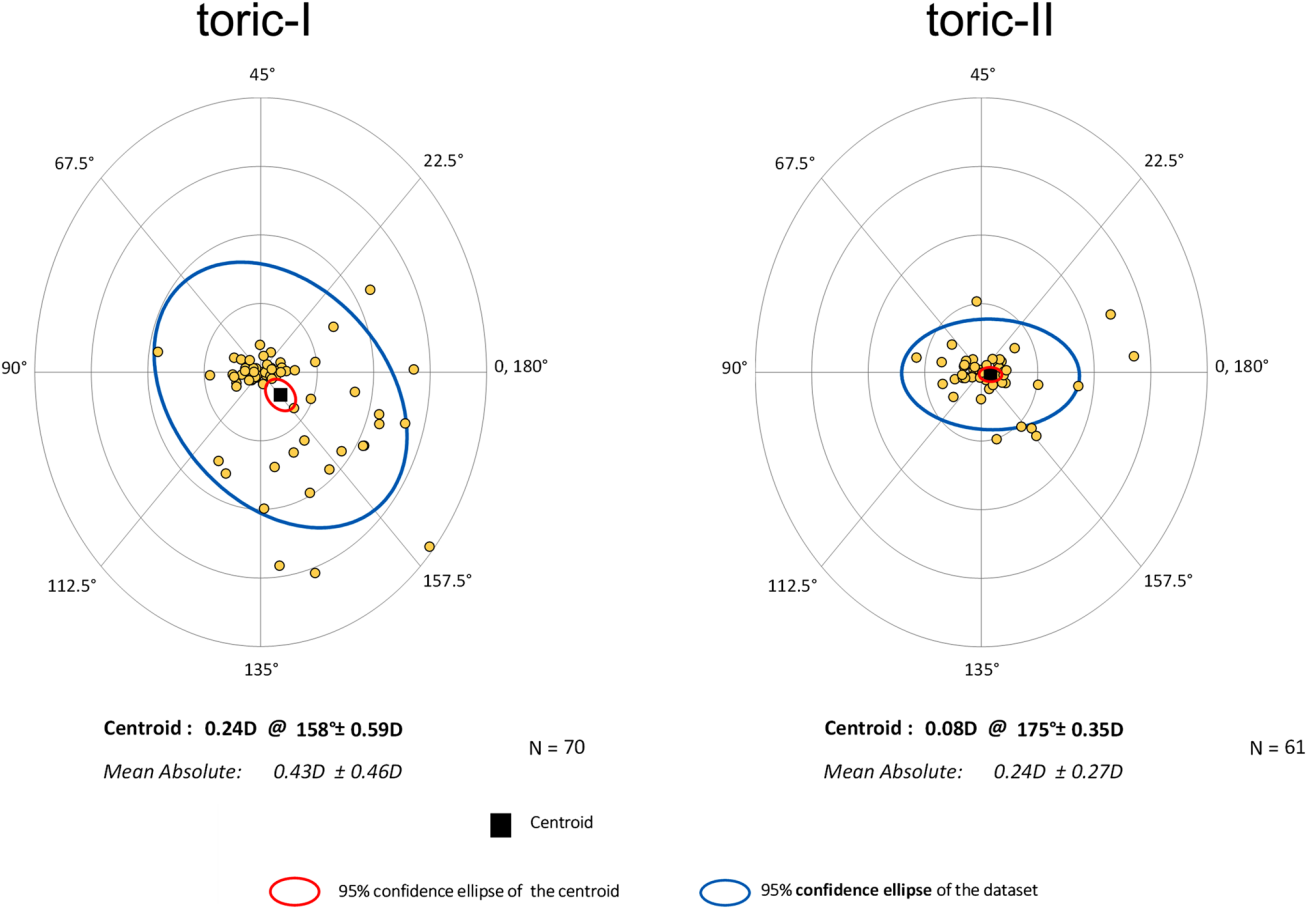
Double-angle plots of refractive astigmatism prediction errors evaluated with centroids, standard deviation of the centroids, and 95% confidence ellipses of the prediction errors. Prediction errors were smaller with toric-II than with toric-I IOLs. Each ring = 0.50 diopters (D).

Surgical results at 3 months postoperatively are presented. The amount of residual manifest astigmatism was significantly smaller in the toric-II IOL than in the toric-I IOL groups (p = 0.018) (Fig. 3). The eyes implanted with toric-II IOL presented significantly smaller amount of axis misalignment from the intended axis than those received toric-I IOL (p = 0.04) (Fig. 4). The percentage of eyes with misalignment > 10° was significantly smaller with toric-II IOLs than with toric-I IOLs (p = 0.0044) (Fig. 5). The prediction errors were smaller in the toric-II IOL than in the toric-I IOL groups (Fig. 6), and their distribution was significantly different between groups (p = 0.043) (Table 2). Postoperative UDVA was compared between the two groups in eyes in which emmetropia was targeted, 61 eyes and 51 eyes in the toric-I and toric-II groups, respectively. The eyes with toric-II IOL tended to show better UDVA (logMAR − 0.11 ± 0.14) than those with toric-I IOL (logMAR 0.024 ± 0.13), but the statistical difference remained marginal (p = 0.057).

**Table 2:** Refractive astigmatism prediction errors.

	Toric-I	Toric-II
≤ 0.25 D	42 eyes (60%)	46 eyes (75%)
≤ 0.50 D	48 eyes (69%)	51 eyes (84%)
≤ 0.75D	53 eyes (76%)	58 eyes (95%)
≤ 1.00 D	60 eyes (86%)	59 eyes (97%)

*D* diopters; Significant difference was found between groups (p = 0.043).

## Discussion

TECNIS toric-II IOL is an improved version of TECNIS toric-I IOL. Both of them are based on the same lens platform, including material, refractive index, design, and configuration. Only difference between these two IOLs is the surface finishing of the haptics; toric-I IOL consists of smooth surface, while the toric-II IOL has frosted surface. In the current clinical study, we found that toric-II IOL had significantly better rotational stability than toric-I IOL, as shown by less amount of axis misalignment from the intended axis, smaller postoperative manifest astigmatism, lower incidence of misalignment > 10°, and less degree of prediction errors. Postoperative UDVA tended to be better in the toric-II than in the toric-I groups, with marginal statistical difference.

Our in vitro experimental study indicated that the unfolding time was significantly shorter with toric-II IOL than with toric-I IOL due to the earlier initial movement of the haptics following ejection of the IOL from the injector. After the haptics started moving, the unfolding process was quite similar and time course was parallel between two IOLs, including complete separation of the haptics from the optic and unfolding to 11 mm in length. When the IOL is getting squeezed while being folded within the cartridge of the injector system, the haptics are in close contact with the optic. After ejected from the cartridge, the haptics are eventually separated from the optic and start moving to recover its shape. It seems that frosted haptics of toric-II IOL contributed to the earlier separation of the haptics from the optic, resulting in faster unfolding of this lens. In general, hydrophobic acrylic IOL has sticky surface and sometimes takes a longer time to unfold than other type of IOLs^14–16^. We assume that the frosted haptics, by offering more surface texture, can reduce surface stickiness of hydrophobic acrylic IOL and expedite unfolding process of the IOL. The quicker unfolding of IOL would lead to earlier and greater contact of the haptics to the capsular bag equator, and can reduce the risk of incomplete unfolding of the haptics at the end of surgery, which is difficult to confirm by direct visualization.

The shorter unfolding time alone, however, cannot explain the improved rotational stability of toric-II IOL, because the unfolding time of this lens is still longer than other hydrophobic acrylic toric IOLs (unpublished data). Although not supported by any concrete evidences, the manufacturer claims that introduction of the frosted haptics is thought to increase the friction between the lens haptics and the capsular bag. Vandekerckhove^17^ compared rotational stability of toric IOLs with identical design and material but different surface treatment, and reported that the IOL with unpolished surface offered better rotational stability probably due to the higher frictional coefficient of its surface. Lens surface adhesiveness is supposed to be an important factor contributing to rotational stability, especially in the early postoperative period, before capsular bag shrinkage occurs^17^. We therefore consider that both faster unfolding and greater friction play roles in improved rotational stability of toric-II IOL over toric-I IOL.

This study has some limitations. First, this was not a prospective study, and thus random assignment of eyes to either model of toric IOLs was not planned. The use of toric-I and toric-II IOLs was not parallel, but sequential. This was because the former IOL was pulled out of the market on the launch of the latter toric IOL, making the use of both IOLs at the same time impractical. Second, the follow-up period of patients was 3 months, and longer clinical results were not evaluated. The positioning of toric IOLs, however, is known to be highly stable after 1 day postoperatively^1^, and therefore we believe that results at 3 months postoperatively are robust enough to extrapolate the longer-term rotational stability of toric IOLs. Third, the mechanism of action underlying improved rotational stability is based on assumptions. Especially, it is difficult to prove that increased friction between the frosted haptics and the capsular bag actually contributed to less misalignment of toric IOLs. There is a possibility that other biomechanical and dimensional factors contribute to the rotational stability of toric IOLs in the capsular bag^18,19^. Further studies are awaited to clarify these points.

In conclusion, we compared the surgical outcomes of two toric IOLs. TECNIS toric II IOL with the frosted haptics showed significantly better rotational stability than its predecessor, probably due to faster unfolding and increased friction with the capsular bag.

## Supplementary Information

Supplementary Legend.

Supplementary Video.

## Data Availability

The datasets generated during and/or analyzed during the current study are available from the corresponding author on reasonable request.
