# Supplementary material for: Influence of frosted haptics on rotational stability of toric intraocular lenses
Source: Sci Rep. 2021 Jul 23;11:15099. doi: 10.1038/s41598-021-94293-3 (PMC8302686; doi:10.1038/s41598-021-94293-3)
Supplement: Supplementary file 1 — Supplementary Legend. [file 41598_2021_94293_MOESM1_ESM.docx]

Legend for supplementary video

The toric-I (left) and toric-II (right) IOLs were ejected in a petri dish filled with balanced salt solution and polar grid of concentric circles with 1-mm steps placed at the bottom. The unfolding process of the IOLs were video recorded. IOLs with same power (+20.0 diopters) and toricity (model 150) were used, and the experiment was carried out at room temperature of 24.0ºC.
